# Embryonic origin and genetic basis of cave associated phenotypes in the isopod crustacean *Asellus aquaticus*

**DOI:** 10.1038/s41598-018-34405-8

**Published:** 2018-11-08

**Authors:** Hafasa Mojaddidi, Franco E. Fernandez, Priscilla A. Erickson, Meredith E. Protas

**Affiliations:** 1 Dominican University of California, 50 Acacia Ave, San Rafael, CA 94901 USA; 2 University of Virginia, 90 Geldard Drive, Charlottesville, VA 22903 USA

## Abstract

Characteristics common to animals living in subterranean environments include the reduction or absence of eyes, lessened pigmentation and enhanced sensory systems. How these characteristics have evolved is poorly understood for the majority of cave dwelling species. In order to understand the evolution of these changes, this study uses an invertebrate model system, the freshwater isopod crustacean, *Asellus aquaticus*, to examine whether adult differences between cave and surface dwelling individuals first appear during embryonic development. We hypothesized that antennal elaboration, as well as eye reduction and pigment loss, would be apparent during embryonic development. We found that differences in pigmentation, eye formation, and number of segments of antenna II were all present by the end of embryonic development. In addition, we found that cave and surface hatchlings do not significantly differ in the relative size of antenna II and the duration of embryonic development. To investigate whether the regions responsible for eye and pigment differences could be genetically linked to differences in article number, we genotyped F2 hybrids for the four previously mapped genomic regions associated with eye and pigment differences and phenotyped these F2 hybrids for antenna II article number. We found that the region previously known to be responsible for both presence versus absence of pigment and eye size also was significantly associated with article number. Future experiments will address whether pleiotropy and/or genetic linkage play a role in the evolution of cave characteristics in *Asellus aquaticus*.

## Introduction

One goal of evolutionary biology research is to understand the genetic basis and evolutionary history of species or populations. These types of studies rely heavily on non-traditional model species which have become easier to study over the last few years due to advances in sequencing technologies and molecular techniques^1–3^. However, an understanding of the developmental biology of the organisms is also necessary to adapt many techniques and to answer questions in emerging model organisms. For example, functional techniques such as RNAi and CRISPR not only rely on genetic information (knowledge of the sequence of genes to be perturbed), but for many species RNAi and CRISPR also require developmental techniques such as embryo injection and the ability to culture and manipulate embryos post injection, ideally *in vitro*. In addition, to functionally perturb a particular phenotype, and to confirm the phenotype is perturbed, one must know when that phenotype arises in development. Moreover, this information can inform comparative transcriptomics at developmental time points to investigate the developmental mechanisms of that phenotype.

Cave animals have many unique features that can offer insight to the developmental and genetic basis of evolutionary change; however, it is difficult to study them as most species have few or no established developmental techniques. Characteristics common to cave dwelling animals include elongation of appendages, loss or reduction of eyes, loss of pigment, enhancement of sensory systems, decreased metabolic rate, and enhanced ability to resist starvation^4^. Because multiple cave animals have evolved similar characteristics, we can compare whether these characteristics have evolved using the same or different mechanisms. In addition, multiple independently evolved populations of a single species can allow for the study of parallel evolution. Therefore, one of the highlights of studying cave animals is that there are many levels at which the evolution of similar characteristics can be studied, between very different species, between closely related species, and between populations of the same species^5^.

The cave animal currently with the most developmental, genetic, and genomic tools is the fish, *Astyanax mexicanus*^6,7^, which contains both cave and surface dwelling populations. The exciting potential and possibilities present in this organism stem from an ability to utilize developmental biology techniques as well as genetic techniques^8–15^. Studies of *A. mexicanus* have been informative about the evolution of this species’ cave characteristics, but to gain a better understanding of evolution in cave animals, one must examine multiple phyla^5^. Fortunately, several recent studies have investigated additional cave-dwelling species. For example, expression of opsin and hedgehog were investigated in surface and cave individuals of the amphipod crustacean *Gammarus minus*^16,17^. Genomes of surface and cave individuals of the fish genus *Sinocylocheilus*, found in China, were sequenced and compared^18^. In addition, transcriptomes of multiple cave organisms such as the cave beetle *Ptomaphagus hirtus*, the fish *Poecilia mexicana*, and the fish *Sinocyclocheilus* have been sequenced and analyzed^19–21^. All of these studies have allowed for a greater understanding of cave biology and evolution. However, many of these studies focus on genomic or genetic resources instead of developmental resources because very few cave animals can be cultured in the lab and, as a result, only rarely can embryonic development be examined.

**Figure 1:**
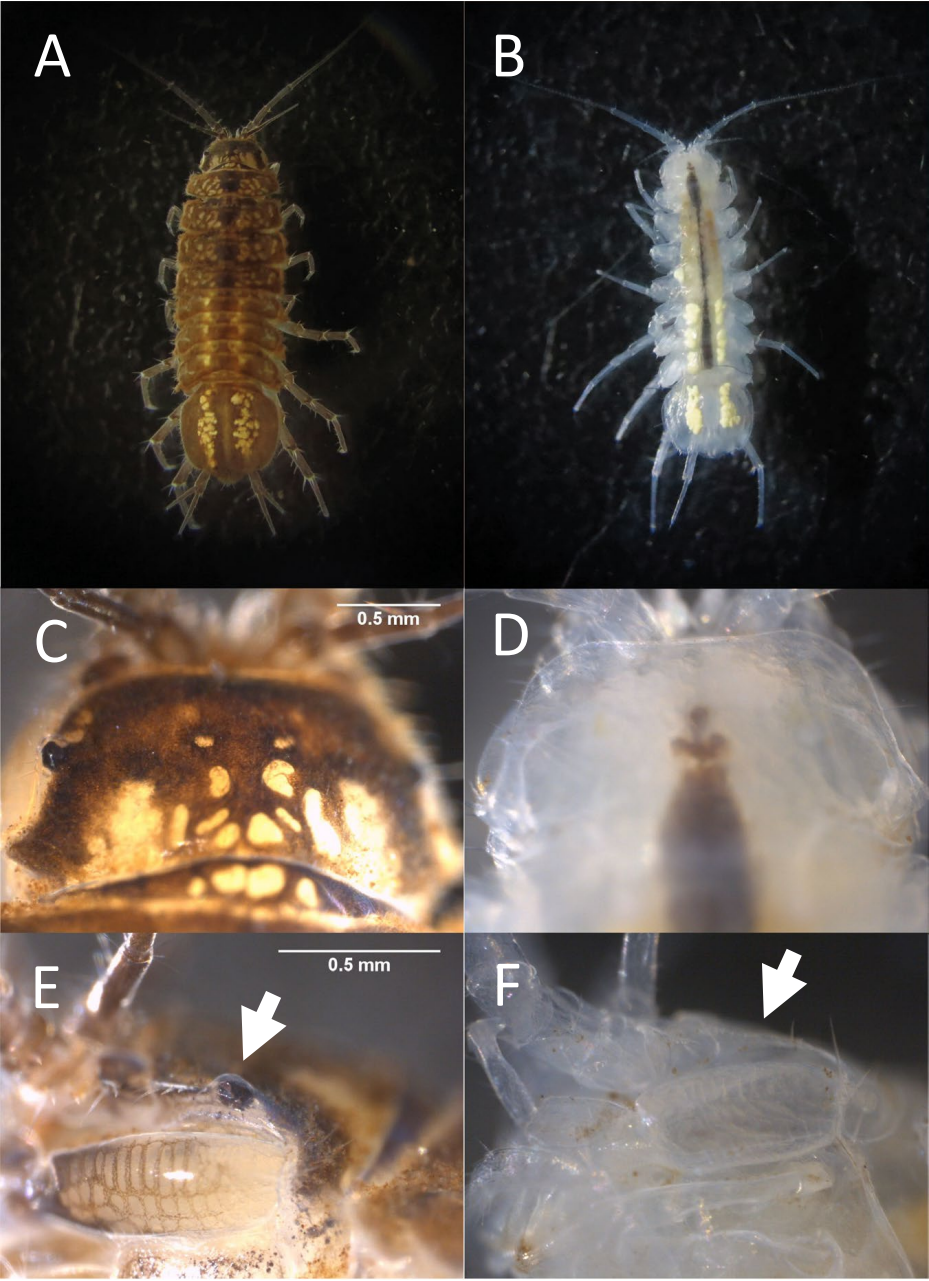
Differences between adult surface and cave individuals. (**A**,**B)** Adult surface individual (**A**) and adult cave individual (**B**). (**C**,**D**) Dorsal views of pigmentation and eye presence in the head of the surface individual (**C**) compared to the head of the cave individual (**D**). (**E**,**F**) Lateral view of left eye (arrow) in a surface adult (**E**) compared to same region in a cave adult showing no eye (arrow) (**F**).

Here, we investigate the embryogenesis of an emerging model, *Asellus aquaticus*, a freshwater isopod crustacean that has both surface and cave forms (Fig. 1). Surface populations are found in freshwater lakes and streams throughout much of Europe, while cave populations are found in several countries including Slovenia and Romania^22–25^. Advantages of this species include the ability to raise the animals in the laboratory using limited space and resources, the existence of multiple, independently evolved cave populations, and the ability to interbreed cave and surface forms^26^.

Extensive morphological characterization has been performed for adults from cave and surface populations of *Asellus aquaticus*, showing reduced or absent ommatidia (units of the eye in arthropods) and reduced or absent pigmentation. In addition, elaboration of sensory structures has also been observed, such as elongated antennae II, in the cave form as compared to the surface form^27,28^. Additional differences seen in Slovenian cave populations include increased relative length of the longest leg, pereopod VII, less numerous spines on two of the legs, pereopod I and pereopod VII, and fewer articles or segments on antenna I^27^. Some genetic resources have been established for *A. aquaticus* including genetic markers and a linkage map^29^. These tools have been used, in conjunction with backcrosses between the cave and surface individuals, to map regions of the genome responsible for eye and pigmentation traits^29^. Several traits have been mapped including presence versus absence of pigmentation, light versus dark pigmentation, red versus brown pigmentation, stellate versus diffuse pigmentation pattern, eye presence versus absence, and eye size^29^. Four genomic regions, so far, were found to be responsible for the aforementioned traits^29^. Two of these regions are responsible for both eye and pigment traits suggesting possible pleiotropy or genetic linkage of derived cave traits. However, it is unknown what the genes responsible for these traits are and whether any of the mapped regions are responsible for other characteristics such as appendage elaboration. Furthermore, it has been shown that the same four regions are responsible for these traits in at least two different cave populations^30^. In addition, transcriptomes of adult individuals have been sequenced and analyzed, placing genes on the linkage map and identifying many more genetic markers^31,32^. Moreover, much information is known about the phylogenetic history of multiple populations of this species^22–25,33^.

Despite these advantages, developmental resources for the cave form of *A. aquaticus* are minimal. Most of the knowledge of the embryonic development of this species is limited to the surface form^34–36^, though a few characters were examined in late stage cave embryos from wild-caught ovigerous females^37^. In the present study, we raise both cave and surface individuals in a common environment to compare embryonic development across cave and surface forms. We selected several characters known to be different in adult cave and surface individuals to examine during embryonic development: eye size, pigmentation, number of flagellar articles on antennae II, and relative size of antennae II. In addition, we intercrossed cave and surface individuals and genotyped F2 hybrids for the four known regions responsible for eye and pigment loss and phenotyped them for antennae II article number. We hypothesized that differences in antennae would be established in embryonic development if elaboration of antennae improves hatchling fitness in the cave environment. Enhancement of antennae has been traditionally suggested as a mechanism for finding food and mates in the dark cave environment^38^, though recently environmental parameters such as water flow and competition with other species are also associated with appendage length^39^. In addition, we hypothesized that reduction of pigment and eyes would occur early in development, similar to other cave organisms^40,41^. And finally, if indeed the reduction of eye and pigmentation and the enhancement of antennae occur in a similar developmental timeframe, we hypothesized there would be some overlap in the genetic regions responsible for eye and pigment loss and those responsible for antennal elaboration.

## Methods

### Animal husbandry

*A. aquaticus* were collected from the Rak channel of Planina Cave (Z), the Rakov Škocjan (RS) surface location, and the Planina Polje (PP) surface location in Slovenia. The Z cave population contains unpigmented and eyeless individuals. The RS surface population contains pigmented and eyed individuals. The PP surface population generally contains pigmented and eyed individuals but we were able to isolate and breed a variant from the PP surface population that developed pigmentation post embryonically, which we used to examine eye development in the absence of pigmentation. Cave and surface animals were kept in the same conditions. The animals were kept in breeding tanks in an incubator set at 12 °C in artificial freshwater^42^ or spring water (Crystal Geyser Alpine Spring Water) and fed decaying leaves of various types, mainly poplar and maple, collected from a local creek (Berkeley, CA) as previously described^29,30^. The incubators did not have internal lights, and animals (both cave and surface) were only exposed to light when the water and food was being changed.

### Embryo collection

Males guard females in amplexus before fertilization occurs. After fertilization occurs, the male releases the female and the female’s brood pouch is ultimately filled with fertilized embryos. Therefore, to collect early embryos, pairs (a male carrying a female underneath him) can be identified and watched until they unpair. To track the morphological changes in both populations during development, embryos were extracted 24 hours after the female was first observed to be carrying embryos. Females with embryos were anesthetized in clove oil (20 µl in 50 mL of artificial freshwater) for about 30 minutes then washed twice with artificial freshwater. Females with embryos were then placed in a petri dish, and the embryos extracted with forceps. Post embryo removal, females were placed into new artificial freshwater for recovery prior to returning to breeding tanks. Embryos were then transferred into new petri dishes with fresh artificial water and placed in the incubator at 12 °C. For all tests, a maximum of two individuals were used per brood, to control for genetic differences specific to different broods.

### Live imaging and tracking of length of embryonic development

Two embryos were assayed from 10 cave broods totaling 20 cave embryos. Two embryos were assayed from 20 surface broods totaling 40 surface embryos. Cave and surface embryos were tracked throughout embryonic development. More surface embryos were used because our lab populations of surface animals are bigger than those of the cave animals. Images were taken twice a week using a Leica S8 Apo stereomicroscope with LAS (Leica) Core software and Image Builder. Images were taken immediately after extraction from the female and continued until hatching. Additional images were taken using a Zeiss Axiovert 40 CFL inverted microscope for higher magnification images of development of the eye.

### Counting of antennal articles

One or two individuals were gathered from different surface and cave broods just prior to hatching to reach a total of 20 individuals per population. Embryos were placed in 100% ethanol (EtOH), and kept at −20 °C. Note, these are different individuals than were used in the developmental time tracking. The number of flagellar articles or segments of the right antennae were counted (both for antenna I and II) using a Zeiss Axiovert 40 CFL inverted microscope.

### Measurement of antennae II length and body size

After counting of antennal articles, we dissected off the right antennae. In three individuals the right antenna was unusable so we used the left antenna. We mounted the antennae on slides in 100% glycerol. Some antennae were destroyed by the mounting process so we analyzed a total of 15 cave individuals and 15 surface individuals. Images were taken using LAS (Leica) core (version 4.9). The LAS Interactive measurement tool was used to measure the length of the antennae. For body size, all legs were dissected off of the individual and the body was put on a slide in one drop of glycerol. A coverslip was put on either side of the body and then another coverslip put on top of those two coverslips. The LAS Interactive measurement tool was used to measure body size.

### Hybrid crosses

We crossed individuals from the surface population, RS, to individuals from the Rak Channel of the Planina Cave (Z). Brother/sister matings were set up from F1 crosses. When a female was observed to have embryos that had proceeded around half way through embryonic development, the embryos were removed as described above. Each hatchling was reared in an individual cup singly with one algae pellet for food until they died or until they reached greater than six months of age. Three quarters of the water was changed every month and one algae pellet was added. Animals were checked every week and when dead were put in ethanol. Therefore, animals were of varying ages when tissue was harvested for genotyping, though phenotyping all occurred at the same time, right at hatching. For the few animals that survived to greater than 6 months of age, tissue was harvested from a leg. As mortality was high and DNA quality was not ideal if the animal had already died, for the final brood 34 (Sup. Table S1) we kept the embryos in the female until hatching, then the animals were treated with clove oil, phenotyped, and immediately harvested for DNA. A total of 85 F2 hybrids were used for this experiment.

### MassArray genotyping

DNA was extracted from either the whole animal or a leg of a large adult animal using QiAamp DNA micro kit (Qiagen). DNA was sent to CD-Genomics for MassArray MALDI-TOF SNP genotyping^43^. Genotyping of multiple markers were performed but ultimately the following four markers were tested for association with antennae article number: *sob* which marks eye presence v. absence, *pax2* which marks red v. orange or brown pigmentation, *pointed* which marks orange v. red or brown pigmentation, and *disconnected* which marks presence v. absence of pigment (Sup. Tables S1 and S2). We have used three of these four markers previously to mark these regions of interest^30^. Previously we had used a marker in *nckx30* to mark the region responsible for orange v. brown pigmentation but we found that this marker did not work well with the MassArray genotyping and therefore used a marker in the gene *pointed* as it is linked to *nckx30*^29^. Ultimately, 40 F2 individuals were successfully genotyped for these genetic markers.

### Sanger sequencing genotyping

A significant number of the F2 individuals were not successfully genotyped using the MassArray SNP genotyping likely because of the low yield and quality of some of our DNA samples. We were able to genotype some of these animals by Sanger Sequencing (Sup. Table S2). PCR conditions used were as previously described^30^. We also sequenced additional individuals with Sanger sequencing that were generated after the MassArray genotyping analysis to bring the final total of genotyped F2 hybrids to 85 (Sup. Table S1).

### Statistical analyses

All analyses were performed in R^44^. To compare the counts of antennal articles between populations, a permutation test was performed using the permTS^44^ package with the default settings. This test was used due to the non-normal, non-continuous data and small sample size. Non-parametric statistics were used for other surface-cave comparisons when appropriate, and linear models were used to regress phenotypes to body size. To test for an association between the four genomic regions responsible for eye and pigment loss and the phenotype of article number in antennae II, a generalized linear model was used to test an additive model with genotypes coded as 0, 1, or 2 based on dosage of the cave allele.

## Results

### Embryonic development of surface-dwelling *Asellus aquaticus*

**Figure 2:**
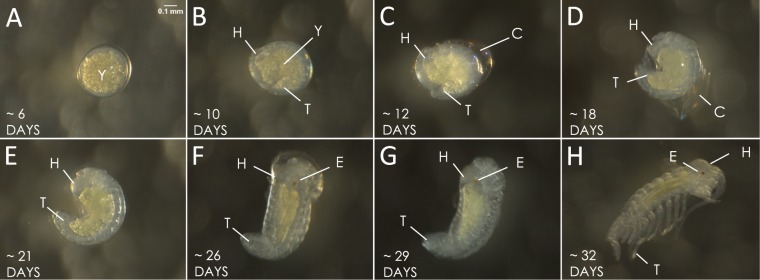
Embryonic development of the surface-dwelling form of *Asellus aquaticus*. (**A**) An embryo at ~6 days post fertilization. (**B**) At ~10 days post fertilization, the head (H) and the tail (T) are distinct. Yolk is (Y). (**C**) Lateral view of embryo at ~12 days post fertilization with the chorion (**C**) starting to shed off. (**D**) Lateral view of embryo now extending while membrane (**C**) continues to be shed. (**E**) Lateral view of embryo with defined head (H) and tail (T) and three membranes remaining. (**F**). At ~26 days, the second membrane is shed, the yolk size is reduced, and eye and head pigmentation (E) is obvious. (**G**) Lateral view after second membrane is shed and there is an increase in eye (**E**) and head pigmentation. (**H**) At ~32 days, the embryo sheds the third membrane and the eye and head have increased pigmentation.

To provide a framework for embryogenesis, we followed embryonic development in surface embryos. Initially, the surface embryo is mostly yolk. After approximately one week, the germ band becomes apparent on the surface of the embryo (Fig. 2A). Ultimately, the germ band elongates and a separation is seen between the anterior and posterior ends (Fig. 2B). The chorion begins to shed shortly after the separation is seen, while the second and third membranes are still intact (Fig. 2C). Three to four weeks post fertilization, embryos then shed the second transparent membrane. During this period, faint red pigmentation is observed in the eye region of the embryo first and then elsewhere on the head of the embryo, the limbs continue to extend, and the body of the embryo becomes straighter and less comma shaped (Fig. 2F). About a month after fertilization, the third membrane is shed and the embryo extends its appendages (Fig. 2H). Soon after, the embryo hatches out of the final membrane and resembles a smaller version of the adult with one fewer pair of legs which have not yet developed.

### Comparison of eye and pigment development in cave and surface embryos

**Figure 3:**
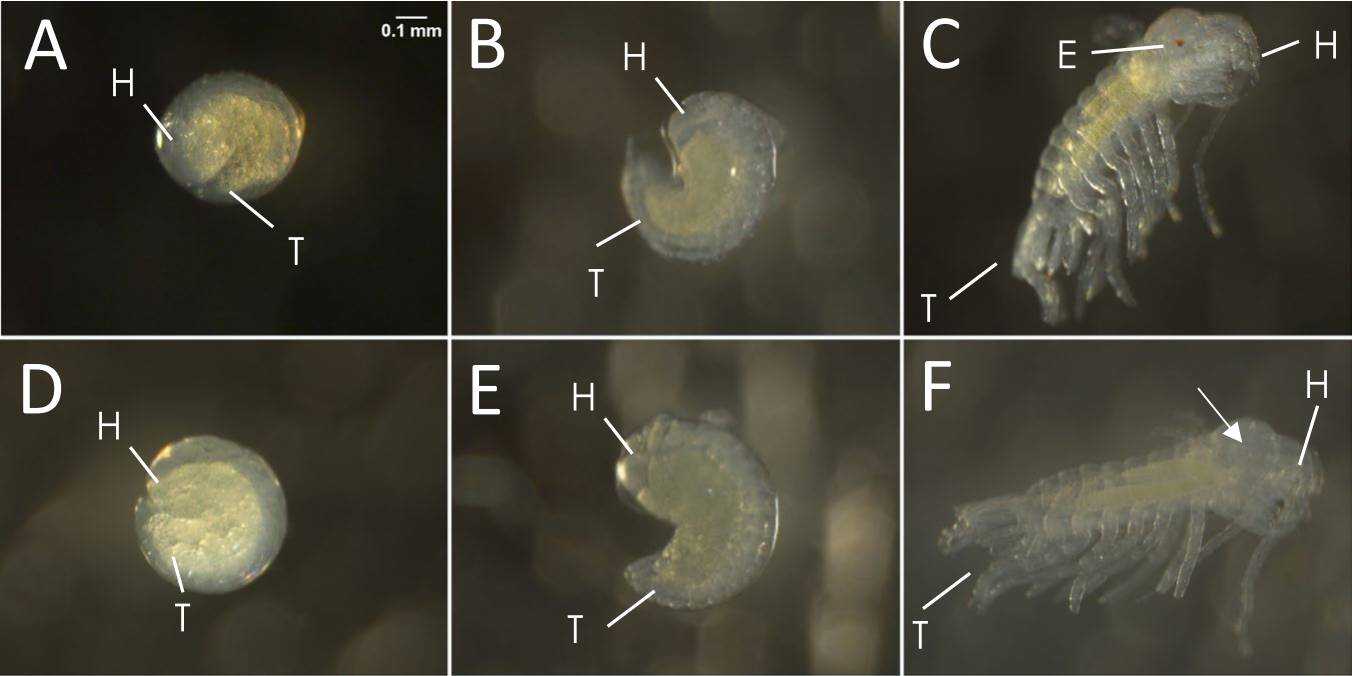
Comparative embryonic development of surface and cave embryos of *Asellus aquaticus*. – Top row: surface embryos (**A**–**C**). (**A**,**D**) The head (H) and tail (T) are now distinct. (**B**,**E**). The embryo is comma-shaped. (**C**) Appendages are freed from the membrane and eye pigmentation (**E**) is observed. Bottom row: cave embryos (**D**–**F**). (**F**) Appendages are freed from the membrane and pigmentation is not observed. Arrow indicates where eye would be seen.

**Figure 4:**
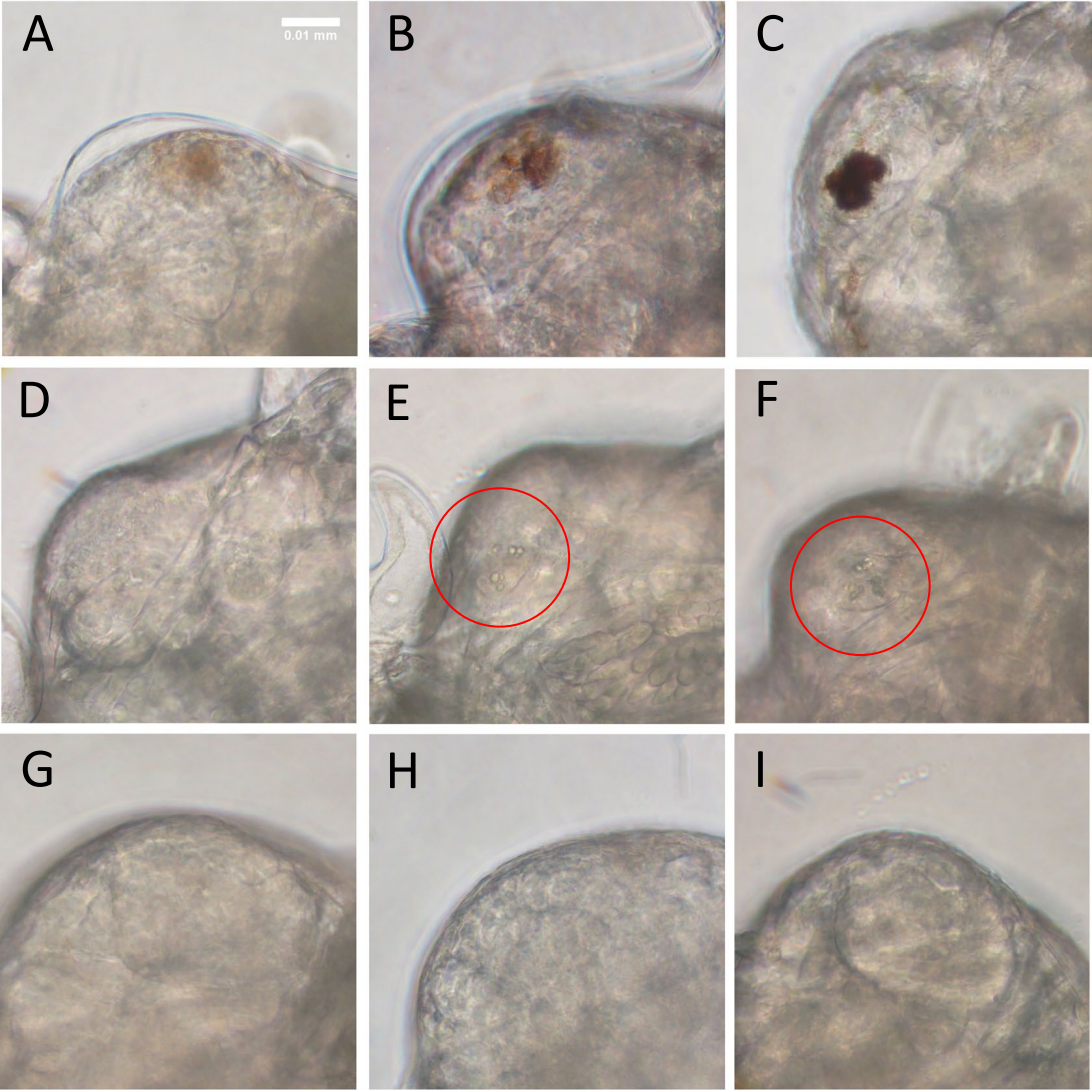
Eye region of surface and cave forms of *Asellus aquaticus*. (**A**–**C**) Surface embryos from RS population at 75% of the way through embryonic development (**A**), 85% of the way through embryonic development (**B**) and 95% of the way through embryonic development (**C**). By the end of embryogenesis (**C**), embryos have 3 pigmented spots (ommatidia are difficult to see because of pigmentation). (**D**–**F**) Surface embryos from a light colored variant from the PP surface population develop small circular fragments (**E**) which then combine into ommatidia (**F**). Fragments and ommatidia shown within the red circles. (**G**–**I**) Cave embryos at the same timepoints show no forming ommatidia. Anterior is to the left.

We examined when embryonic development differed among cave and surface embryos. The development of cave embryos was identical to that described above for the surface embryos until pigmentation started developing in the surface individuals (Fig. 3); the surface embryos developed pigmentation in the eyes but the cave embryos did not. As embryos approached hatching, the surface embryos became more pigmented both in the eye region and throughout the body but the cave embryos never developed pigmentation (Figs. 3 and 4).

Next, we examined the formation of ommatidia (units of the eye in arthropods) in embryos of the two forms. Surface adults possess four ommatidia^45^ but cave adults lack ommatidia or have degenerate ommatidia^46^. Three pigmented spots were seen developing in surface embryos from Rakov Škocjan (RS) (Fig. 4A–C). The eye pigmentation in the surface form made it difficult to visualize forming ommatidia, and therefore a naturally occurring surface variant with light pigmentation from the adjacent surface population Planinska Polje (PP) was also tracked (Fig. 4D–F). In this light surface variant, three ommatidia are clearly seen developing with initial formation of many small circles that combine to form three larger circles (Fig. 4D–F). Adults have four ommatidia and therefore the fourth ommatidium must develop post embryonically. In contrast, cave embryos did not show evidence of developing ommatidia at the same stages (Fig. 4G–I).

### Comparison of relative antennal size in surface and cave hatchlings

**Figure 5:**
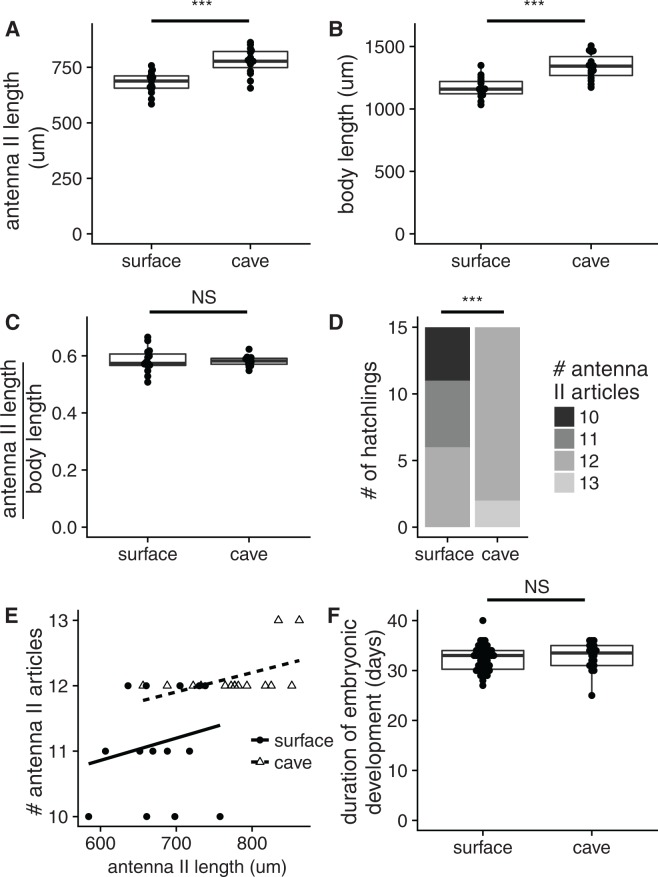
Antenna II comparisons and embryonic development comparisons in cave and surface *Asellus aquaticus*. (**A**) Absolute antenna II size comparing 15 surface hatchlings and 15 cave hatchlings. Two tailed Mann-Whitney U-test, *P* = 8.979 × 10^−5^. (**B**) Absolute body size comparing 15 surface hatchlings and 15 cave hatchlings. Two tailed Mann-Whitney U-test, *P* = 5.619 × 10^−5^. (**C**) Relative antenna size comparing 15 surface hatchlings and 15 cave hatchlings. Wilcoxon Rank Sum *P* = 0.9349. (**D**) Article count in right antenna II of 20 surface hatchlings and 20 cave hatchlings of *Asellus aquaticus*. PermTS *P* = 1.482 × 10^−5^. (**E**) # of articles versus antennal length in cave and surface hatchlings. (**F**) Duration of embryonic development in 40 surface and 20 cave embryos. Mann-Whitney U-test, *P* = 0.2622. ***P < 1 × 10^−4^, NS: *P* > 0.05.

We also investigated another common cave characteristic, elaboration of antennae, which might allow individuals of the cave population to find one another and/or food in the dark cave environment^38^. We compared the size of antenna II in 15 cave and 15 surface hatchlings (Fig. 5A; Sup. Table S3). We found that the size of antenna II is significantly greater in cave hatchlings (average of 777.15 µm) compared to surface hatchlings (average of 680.64 µm) (two tailed Mann-Whitney U-test, *P* = 8.979 × 10^−5^). In addition, we found that the body size was significantly greater in cave hatchlings (average of 1337.53 µm) compared to surface hatchlings (average of 1169.17 µm, Fig. 5B; Sup. Table S3, two tailed Mann-Whitney U-test, *P* = 5.619 × 10^−5^). Body size is moderately correlated to antenna II length in the surface hatchlings (*P* = 0.06), and this correlation is significant in the cave hatchlings (*P* = 6 × 10^−7^). When combining both cave and surface hatchlings, antenna II length is significantly correlated to body size (*P* = 0.008; Sup Fig. S1A), but there is no difference in antenna II length between cave and surface hatchlings after correcting for size differences with a linear model (*P* = 0.2). Likewise, the relative length of antenna II (antennal length divided by body length), was not significantly different between cave and surface hatchlings (Fig. 5C) (two tailed Mann-Whitney U-test, *P* = 0.9349).

### Comparison of antennal article number in surface and cave hatchlings

In *A. aquaticus* adults, the number of antennal articles in antennae I is smaller for cave populations as compared to surface populations, except for the cave population in this particular study. However, the number of antennal articles in antennal II is greater in all cave populations examined compared to surface populations^25,27,28^ (Prevorčnik, pers. comm.). Article number for all antennae were counted for 20 surface and 20 hatchlings (Fig. 5D; Sup. Table S3). The article number for antennae I did not vary between cave and surface hatchlings and was four articles for all individuals. The number of articles on antennae II is significantly greater among cave hatchlings, average of 12.2 articles, compared to surface hatchlings, average of 11.25 (one-tailed permutation test, p = 1.482 × 10^−5^). However, article number is not significantly greater after adjusting for body size in a linear model (*P* = 0.12, Supplemental Fig. S1B). We tested whether the increase in article number was independent of increased antenna length using a linear model. We found that cave individuals have more articles after adjusting for antennal length (*P* = 0.0345, Fig. 5E).

### Comparison of duration of embryonic development in cave and surface forms

Prolonged embryogenesis is also a common cave characteristic. Our null hypothesis was that there is no difference in duration of embryonic development in cave and surface individuals. To test the hypothesis, we compared the duration of embryonic development in 20 cave embryos and 40 surface embryos (Fig. 5F; Sup. Table S4). The duration of embryonic development in cave and surface embryos is not significantly different (~32 days, two tailed Mann-Whitney U-test, *P* = 0.2622).

### Genetic basis of article number in F2 crosses

**Figure 6:**
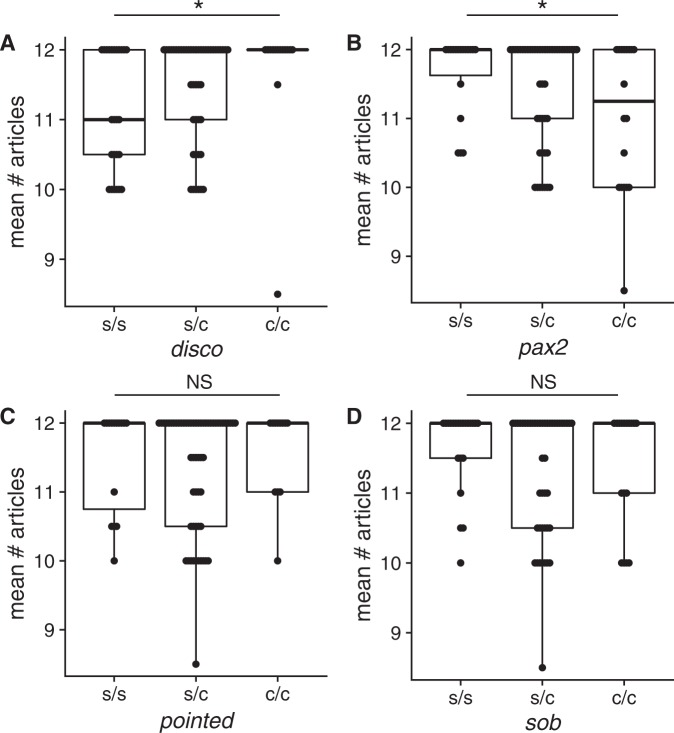
Association of 4 genomic regions responsible for eye and pigment differences with the mean article number of antenna II. (**A**,**B**) *Disconnected* and *pax2* markers are significantly associated with article number but with opposite allelic effects (*P* = 0.02 for each, [*]). (**C**,**D**) *Pointed*, and *sob* markers are not significantly associated with article number (*P* = 0.21 and 0.87, respectively [NS]). s/s indicates two copies of the surface allele, s/c one copy of the surface allele and one copy of the cave allele, and c/c two copies of the cave allele.

Previous work had mapped regions responsible for eye and pigment loss in the Pivka Channel of Planina Cave^29^. Subsequent work found that the same four regions were also responsible for eye and pigment loss in the Rak Channel of Planina Cave which is the population that we are currently examining^30^. To investigate whether the previously mapped regions responsible for eye and pigment loss were also responsible for the genetic variation in article number, we genotyped 85 F2 individuals for each of four regions responsible for eye and/or pigment loss (Fig. 6). We were not able to measure body or antennal size because to make those measurements, we would have had to sacrifice the animal at hatching stages and would not be able to gather enough DNA from those individuals for the genotyping. We measured the number of articles in the right and left antennae and averaged them (if both were present and unbroken) and tested for the effect of each copy of a cave allele with a linear model. The cave allele of the region responsible for presence versus absence of pigmentation and eye size (marked by *disconnected*) significantly increased article number (Fig. 6A, linear model effect = 0.30, t = 2.31, *P* = 0.023). Surprisingly, *pax2* (marking the region responsible for red v. orange or brown pigmentation) also had a significant effect but in the opposite direction: the cave allele reduced article number (Fig. 6B, effect = −0.32, t = −2.35, *P* = 0.021). We did not see a significant association for *sob* (marking the region responsible for eye presence v. absence and stellate v. diffuse pigmentation, *P* = 0.21) or *pointed*, (marking the region responsible for orange v. red or brown pigmentation, *P* = 0.87). While we note that the *disco* and *pax2* effects do not pass a strict Bonferroni-corrected *P*-value threshold of 0.0125, the presence of two out of four markers with effects on article number strongly suggest that there is a genetic basis for this evolved difference between cave and surface individuals.

## Discussion

Understanding when in development morphological differences are established can provide insight into how they have evolved. We compared embryonic development in surface and cave individuals of *A. aquaticus* examining eye, pigment, and antennal phenotypes. Our hypothesis was supported in that we found that eye, pigment, and article number differences on antenna II were established by the end of embryonic development, though article number differences were at least in part caused by differences in body size. However, counter our prediction, we found that the increased relative length of antennae II in the cave form was not significantly different between cave and surface individuals by the end of embryonic development. We also found that one of the four regions previously shown to be responsible for eye and pigment loss in the cave form was significantly associated with antennal article number increase.

Previously, it was shown that late embryos from wild-caught ovigerous females were not pigmented in either the eye or body^37^. However, these embryos were not tracked through the entirety of embryonic development so it was not known if the embryos were pigmented at some point and then lost their pigmentation or whether starting embryonic development in different environments (cave versus surface environment) could have affected pigmentation development. Our studies, examining the entirety of embryonic development in the same environmental conditions, showed that the cave embryos were not pigmented at any point. Therefore, it seems that the loss of pigmentation in adult animals stems from a lack of formation of pigmentation rather than development and subsequent degeneration of pigmentation. This is similar to what is seen in albino populations of the cavefish *A. mexicanus* where pigment never is present^41^.

In addition, to the absence of pigmentation, ommatidia are never seen during the embryogenesis of the cave form of *A. aquaticus*. Studies of a cave amphipod crustacean, *Niphargus virei*, similarly showed that defects in eye development were established in embryogenesis^40^. In the cavefish, *A. mexicanus*, however, the eye begins to develop similarly to surface embryos and then degenerates over time^47^. There are several possible interpretations for the absence of ommatidia. First, it is possible that the eye does begin to form in *A. aquaticus*; photoreceptors could develop internally and then mechanisms similar to those present in *A. mexicanus*, such as progressive apoptosis and degeneration, result in the eyeless phenotype. There is some evidence for this from studies performed on adult cave *A. aquaticus* which show degenerate eye regions described as eye nuclei, though it is unclear whether these eye nuclei are photoreceptors or some other cell type^46^. Another alternative is that the formation of the eye halts very early in development and then is almost completely lost in *A. aquaticus*, aside from the eye nuclei described by Kosswig and Kosswig^46^.

The existence of a degenerate eye can be tested by future studies using antibody staining, *in-situ* hybridization, RT-qPCR, and transcriptomics to investigate candidate genes in eye development and eye structure. If an eye starts to form internally in the cave form, we would expect to see expression of candidate genes involved in eye development such as *pax6, sine oculis*, and *eyes-absent*. Studies of a different cave population of *Asellus aquaticus*, from Hungary, have shown expression of opsins and genes involved in phototransduction^33^. Other cave animals with either absent or reduced eye structures, such as the cave beetle, *Ptomaphagus hirtus*, similarly showed expression of phototransduction genes and structural photoreceptor genes^48^.

Our studies regarding the antennal phenotype showed that antenna II of cave hatchlings did indeed have, on average, one more article than surface hatchlings though antenna I did not differ between the two forms. The relative length of antenna II, though, did not differ significantly between the cave and surface form. Therefore, though the difference in article number was established during embryonic development, the adult difference in relative length must be established post embryonically. In addition, though the difference in article number between cave and surface hatchlings appears to be established during embryonic development, the difference is relatively subtle (the surface antenna II has on average one less article than the cave antenna II upon hatching (Supplementary Table S3)) likely due to the relatively small number of articles in a hatchling. As adults, antenna II from male surface individuals had on average 65 articles and cave adults had on average 95 articles^27^. Therefore, although the difference is established by the end of embryonic development, the difference is accentuated throughout postembryonic development. We have currently focused on comparisons at hatching because many complications exist with examining individuals postembryonically. For example, the animals vary greatly in size and growth rate differences (even within populations). In addition, males have longer antennae than females but it is not possible to identify sex until individuals have reached near adult size. Furthermore, antennae are very likely to break and be in the process of regenerating so it can be difficult to obtain individuals with complete antennae. Further studies will examine the timeline of antennal article number differences and relative length differences between cave and surface forms by tracking and raising many individuals in isolation to minimize antennal breakage and resulting regeneration. In addition, it will be important to examine whether different genetic changes are responsible for embryonic and postembryonic differences in article numbers of the antennae in cave and surface individuals.

There are several possible explanations as to why, for the individuals we compared, the body size, antennae length, and number of articles in antennae II in the cave form was larger than that of the surface form. First, body size and antennae morphology could be correlated to the size of the mother, with bigger mothers producing larger hatchlings with larger antennae; this would argue that the difference in article number is due to mother size rather than genetic differences between cave and surface population. However, in the surface form of *Asellus aquaticus*, the size of the female was not shown to correlate with embryo size^49,50^, though the size of the female is correlated with embryo size in some species^51,52^. Additionally, when examining F2 broods with 5 or more hatchlings, we saw that all of the broods contained variation in article number (Sup. Table S5). If female size played a major role in the number of articles of her offspring, we would expect a single females’ offspring to be more homogeneous in phenotype. Alternatively, cave hatchlings may be larger than surface hatchings due to genetic differences between the populations, and increased antennal length and article number may be caused by this increased body size. Our experiments with F2 individuals showed a genetic association between the markers in *disconnected* and *pax2* and the phenotype of article number (Fig. 6A and B) showing that there is a genetic basis to interpopulational differences to article size in antenna II. However, as we were unable to measure body length in the F2s for technical reasons, we cannot rule out the possibility that the effect of the *pax2* or *disconnected* regions on article number were mediated by an effect of these regions on body size. Furthermore, regressions of article number to antenna length show that cave and surface hatchlings with similarly sized antennae on average had nearly one more antennal article; therefore, a change in article number relative to antenna length occurs in addition to increased antenna length and increased body size (Fig. 5E). We propose that the difference in article number between cave and surface hatchlings is due to genetic differences in the populations rather than maternal size. These genetic changes likely both increase body size and antennal length, and they affect the number of articles relative to antenna length.

Another possible reason that the number of articles could be greater in the cave form is that the cave form could have a long duration of development and as a result be bigger and have more antennal articles upon hatching. However, we found there was a similar range of days to the end of development in cave and surface individuals (25 to 40 days) (Fig. 5F; Supplementary Table S4). This range is similar to a previous study that examined the length of embryonic development in the cave form and showed that cave embryos took between 19 and 47 days to develop; however, that study used embryos from eight broods, which were raised at different temperatures^53^. Future work will examine whether cave individuals grow faster than surface individuals during embryonic development.

Because differences in article number, eye, and pigment were all established embryonically, we tested whether there could be commonalities between the genomic regions responsible for these phenotypes. We found that the same region responsible for presence versus absence of pigment and eye size differences^29,30^ was also significantly associated with antennal article number variation. This result mirrors multiple other studies where multiple traits map to the same region (reviewed in^54–58^). In some cases, a single pleiotropic gene is responsible for different phenotypes but in others, multiple linked genes are responsible^59–64^. We cannot yet differentiate between these possibilities for *A. aquaticus* but further studies identifying the actual gene(s) responsible will illuminate whether pleiotropy is playing a role in the evolution of multiple traits in this species.

In conclusion, we found that pigmentation, eye size, and antennal article number differences were established by the end of embryonic development. However, no significant difference was seen in the number of articles of antennae I and time of embryonic development. Furthermore, we found that the regions responsible for presence versus absence of pigmentation and red versus orange and brown pigmentation were also significantly associated with a difference in number of antennal articles.

Our results provide the framework for studying the developmental biology of cave and surface populations of *A. aquaticus*. This is important as this species has all of the necessary characteristics of a model organism, which is extremely rare for a cave-dwelling species, and combined with the information already harnessed from the cavefish *Astyanax mexicanus*, will provide a better understanding of the evolution of cave characteristics. We have established methods to rear cave and surface individuals of *A. aquaticus* in the lab, interbreed the forms, and examine embryonic development in both forms. Remaining challenges include rearing large numbers of animals in the lab to allow for large scale analyses, working with early embryos and establishing injection protocols, and establishing methods for rearing additional cave populations in the laboratory. As these challenges are met, a next step is isolating early embryos and injecting them to establish functional techniques such as CRISPR. In addition, because we have identified when differences between eye, pigment and appendage length occur we will now be able to pinpoint when embryonic samples should be sequenced for comparative transcriptomics. The goal of embryonic comparative transcriptomics will be to identify genes and pathways responsible for the observed differences between cave and surface forms. Finally, our work provides a stepping stone to investigate the role of pleiotropy in this system. Therefore, we have now established the necessary initial steps for the successful establishment of *Asellus aquaticus* as a developmental model system to understand the evolution of cave characteristics.

## Electronic supplementary material

Supplementary Information

Supplementary Table 1

## Data Availability

All data generated or analyzed during this study are included in this published article and its Supplementary Information files.
