# Supplementary material for: Embryonic origin and genetic basis of cave associated phenotypes in the isopod crustacean Asellus aquaticus
Source: Sci Rep. 2018 Nov 8;8:16589. doi: 10.1038/s41598-018-34405-8 (PMC6224564; doi:10.1038/s41598-018-34405-8)
Supplement: Supplementary file 1 — Supplementary Information [file 41598_2018_34405_MOESM1_ESM.docx]

**Embryonic origin and genetic basis of cave associated phenotypes in the isopod crustacean *Asellus aquaticus***

Hafasa Mojaddidi^1^, Franco Fernandez^1^, Priscilla A. Erickson^2^, and Meredith E. Protas^1^

^1^Dominican University of California, 50 Acacia Ave San Rafael, CA 94901

^2^University of Virginia, 90 Geldard Drive, Charlottesville, VA 22903

correspondence to meredith.protas@dominican.edu

**Supplementary Table S2: Primers used for Mass Array and Sanger sequencing genotyping. MA= Mass Array primers and S=Sanger sequence primers. Sanger sequence primers used for *disconnected*, *pax2*, and *sob* are previously described (Re et al., 2018). SNP used is listed in brackets with the cave allele first and surface allele second.**

| **gene** | **forward primer** | **reverse primer** | **extension primer** | **SNP** |
| --- | --- | --- | --- | --- |
| ***disconnected* (MA)** | 5’ACGTTGGATGTTTCCCAGGTCAGTTTGGAG3’ | 5’ACGTTGGATGATGCTTATGCAAGTCAGCGG3’ | 5’TTTGGAGGCATAAATCCTCTAAC3’ | GGCAACCC[T/A]GTTAGAGG |
| ***pointed* (MA)** | 5’ACGTTGGATGACTCCAGACTCAAAACCACC3’ | 5’ACGTTGGATGGGACCGCCGTTATTATTTTG3’ | 5’TCCGCAAAACCACCATCAGGCTTA3’ | ATCAGGCTTA[C/T]TATGCCCTAA |
| ***pointed* (S)** | 5’CTTTTACGGTCTCCCTGAAAAGTA3’ | 5’CTGTTTCTTGTCCGTCAGAAGTT3’ | NA | same as above |
| ***pax2* (MA)** | 5’ACGTTGGATGCACTGTTTTTTTGTGGATAGC3’ | 5’ACGTTGGATGGAGGAAGGAGTGCTGGTTTG3’ | 5’TTTTTGTGGATAGCACTTAT3’ | TNATAGCACT[--------------------/TTTACTACGTTGATAGCACT]GTTTTTTTGTG |
| ***sob* (MA)** | 5’ACGTTGGATGAGGATCTTATGGACAGCCAG3’ | 5’ACGTTGGATGTCCGCCAGGTATATTCACAG3’ | 5’CAGGGTTCGACTCTG3’ | TTCGACTCTG[G/A]CAAAATCCTT |

**Supplementary Table S3: Number of articles, antennal length, and body size for cave and surface hatchlings.** The right antenna was used except in cases when it was damaged and then the left antenna was used instead. For five additional individuals, article number data was available (shown in Figure 5D) though the other measures were not available.

| Sample name | # art antenna II | Body size (µm) | Antenna size (µm) | Relative length | Sample name | # art antenna II | Body size (µm) | Antenna size (µm) | Relative length |
| --- | --- | --- | --- | --- | --- | --- | --- | --- | --- |
| surface 1 | 12 | 1348.49 | 738.25 | 0.55 | cave 1 | 12 | 1460.04 | 825.23 | 0.57 |
| surface 2 | 11 | 1148.03 | 606.59 | 0.53 | cave 2 | 12 | 1305.24 | 777.24 | 0.60 |
| surface 3 | 12 | 1233.41 | 704.80 | 0.57 | cave 3 | 13 | 1469.09 | 834.72 | 0.57 |
| surface 4 | 10 | 1160.45 | 757.73 | 0.65 | cave 4 | 12 | 1378.68 | 817.53 | 0.59 |
| surface 5 | 11 | 1168.87 | 717.49 | 0.61 | cave 5 | 12 | 1464.98 | 852.39 | 0.58 |
| surface 6 | 11 | 1206.50 | 688.24 | 0.57 | cave 6 | 13 | 1505.68 | 862.251 | 0.57 |
| surface 7 | 12 | 1273.20 | 730.36 | 0.57 | cave 7 | 12 | 1346.16 | 793.18 | 0.59 |
| surface 8 | 12 | 1059.57 | 705.03 | 0.67 | cave 8 | 12 | 1172.468 | 688.053 | 0.59 |
| surface 9 | 10 | 1033.35 | 584.39 | 0.57 | cave 9 | 12 | 1308.29 | 776.922 | 0.59 |
| surface 10 | 11 | 1158.84 | 669.09 | 0.58 | cave 10 | 12 | 1197.04 | 656.154 | 0.55 |
| surface 11 | 12 | 1253.17 | 636.33 | 0.51 | cave 11 | 12 | 1342.8 | 772.325 | 0.58 |
| surface 12 | 10 | 1112.03 | 660.99 | 0.59 | cave 12 | 12 | 1287.24 | 722.312 | 0.56 |
| surface 13 | 10 | 1130.20 | 698.11 | 0.62 | cave 13 | 12 | 1226.978 | 764.517 | 0.62 |
| surface 14 | 11 | 1149.18 | 651.89 | 0.57 | cave 14 | 12 | 1248.44 | 732.611 | 0.59 |
| surface 15 | 12 | 1102.28 | 660.38 | 0.60 | cave 15 | 12 | 1349.78 | 781.81 | 0.58 |
| average | 11.13 | 1169.17 | 680.64 | 0.58 | average | 12.13 | 1337.53 | 777.1499333 | 0.58 |

**Supplementary Table S4: Duration of embryonic development in cave and surface individuals**

|  | **Duration of embryonic development (in days)** |
| --- | --- |
| surface 1 | 27 |
| surface 2 | 28 |
| surface 3 | 32 |
| surface 4 | 30 |
| surface 5 | 30 |
| surface 6 | 31 |
| surface 7 | 29 |
| surface 8 | 29 |
| surface 9 | 31 |
| surface 10 | 30 |
| surface 11 | 32 |
| surface 12 | 32 |
| surface 13 | 31 |
| surface 14 | 30 |
| surface 15 | 30 |
| surface 16 | 30 |
| surface 17 | 31 |
| surface 18 | 30 |
| surface 19 | 32 |
| surface 20 | 29 |
| surface 21 | 29 |
| surface 22 | 31 |
| surface 23 | 31 |
| surface 24 | 34 |
| surface 25 | 33 |
| surface 26 | 33 |
| surface 27 | 36 |
| surface 28 | 35 |
| surface 29 | 35 |
| surface 30 | 34 |
| surface 31 | 33 |
| surface 32 | 34 |
| surface 33 | 34 |
| surface 34 | 34 |
| surface 35 | 35 |
| surface 36 | 36 |
| surface 37 | 36 |
| surface 38 | 33 |
| surface 39 | 33 |
| surface 40 | 34 |
| surface 41 | 33 |
| surface 42 | 33 |
| surface 43 | 33 |
| surface 44 | 33 |
| Surface 45 | 34 |
| surface 46 | 35 |
| surface 47 | 35 |
| surface 48 | 33 |
| surface 49 | 34 |
| surface 50 | 40 |
| cave 1 | 25 |
| cave 2 | 32 |
| cave 3 | 31 |
| cave 4 | 31 |
| cave 5 | 30 |
| cave 6 | 30 |
| cave 7 | 31 |
| cave 8 | 32 |
| cave 9 | 35 |
| cave 10 | 34 |
| cave 11 | 34 |
| cave 12 | 33 |
| cave 13 | 36 |
| cave 14 | 34 |
| cave 15 | 33 |
| cave 16 | 35 |
| cave 17 | 35 |
| cave 18 | 36 |
| cave 19 | 34 |
| cave 20 | 36 |
| **Average surface** | **32.4** |
| **Average**  **cave** | **32.85** |

**Supplementary Table S5: Number of articles of antennae II in F2 hybrids crossed between surface and cave populations.** Only broods with 5 or more hatchlings were used for this comparison. The number of articles was averaged between right and left antennae II. If the average is 11.5, the number is round upwards to 12. Note- not all of these individuals were successfully genotyped so not all are present in Supplementary Table S1

|  | 9 articles | 10 articles | 11 articles | 12 articles | 13 articles |
| --- | --- | --- | --- | --- | --- |
| **brood 1** | 0 | 1 | 4 | 6 | 0 |
| **brood 2** | 0 | 3 | 2 | 5 | 0 |
| **brood 6** | 0 | 3 | 3 | 6 | 0 |
| **brood 7** | 0 | 0 | 2 | 5 | 0 |
| **brood 8** | 0 | 3 | 1 | 4 | 0 |
| **brood 11** | 0 | 0 | 1 | 3 | 1 |
| **brood 18** | 0 | 0 | 1 | 6 | 0 |
| **brood 17** | 0 | 2 | 1 | 3 | 0 |
| **brood 24** | 1 | 0 | 1 | 6 | 0 |
| **brood 25** | 0 | 0 | 2 | 4 | 0 |
| **brood 30** | 0 | 1 | 4 | 3 | 0 |
| **brood 34** | 0 | 4 | 3 | 18 | 0 |

**Supplementary Figure S1:** **Antenna II length and # of antenna II articles versus body size.** Colored lines indicate regression lines within each population; black dashed line is the regression of the combined populations. A. Antenna II length versus body size. Within the surface population, body size is moderately correlated to antenna length (*P* = 0.06), and within the cave population, body size is significantly correlated to antenna length (*P* = 6 x 10^-7^). When combining individuals from both populations, antenna length is significantly correlated to body size (*P* = 0.008) and there is no effect of population (*P* = 0.2). B. # of antenna II articles versus body size. Within the surface population, body size is moderately correlated with article number (*P* = 0.05), and within the cave population, body size is significantly correlated to article number (*P* = 0.02). Across both populations, article number is significantly correlated to body size (*P* = 0.0008) and there is no effect of population (*P* = 0.12).


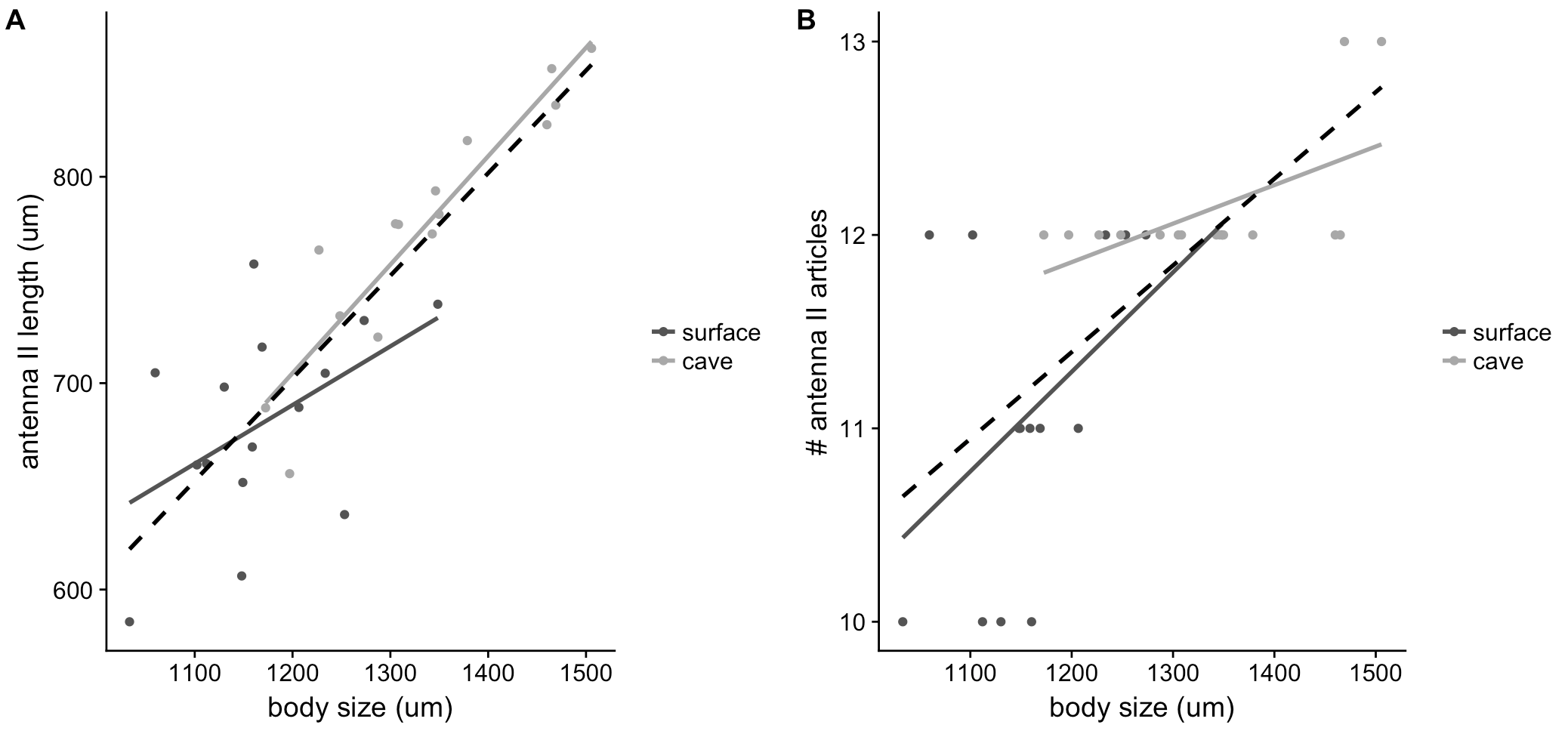


References:

Re, C. *et al*. Common genetic basis of eye and pigment loss in two distinct cave populations of the isopod crustacean *Asellus aquaticus*. *Integr. Comp. Biol.* **58**, 421-430 (2018).
